# EEG-Based Control for Upper and Lower Limb Exoskeletons and Prostheses: A Systematic Review

**DOI:** 10.3390/s18103342

**Published:** 2018-10-07

**Authors:** Maged S. AL-Quraishi, Irraivan Elamvazuthi, Siti Asmah Daud, S. Parasuraman, Alberto Borboni

**Affiliations:** 1 Smart Assistive and Rehabilitative Technology (SMART) Research Group & Department of Electrical and Electronic Engineering, Universiti Teknologi PETRONAS, Bandar Seri Iskandar 32610, Malaysia; eng.mgd@gmail.com (M.S.A.-Q.); sitiasmah.daud@utp.edu.my (S.A.D.); 2 Faculty of Engineering, Thamar University, Dhamar 87246, Yemen; 3 School of Engineering, Monash University Malaysia, Bandar Sunway 46150, Malaysia; s.parasuraman@monash.edu; 4 Mechanical and Industrial Engineering Department, Universita degli Studi di Brescia, Via Branze, 38-25123 Brescia, Italy; alberto.borboni@unibs.it

**Keywords:** EEG, brain machine interface, lower limb exoskeleton, upper limb exoskeleton

## Abstract

Electroencephalography (EEG) signals have great impact on the development of assistive rehabilitation devices. These signals are used as a popular tool to investigate the functions and the behavior of the human motion in recent research. The study of EEG-based control of assistive devices is still in early stages. Although the EEG-based control of assistive devices has attracted a considerable level of attention over the last few years, few studies have been carried out to systematically review these studies, as a means of offering researchers and experts a comprehensive summary of the present, state-of-the-art EEG-based control techniques used for assistive technology. Therefore, this research has three main goals. The first aim is to systematically gather, summarize, evaluate and synthesize information regarding the accuracy and the value of previous research published in the literature between 2011 and 2018. The second goal is to extensively report on the holistic, experimental outcomes of this domain in relation to current research. It is systematically performed to provide a wealthy image and grounded evidence of the current state of research covering EEG-based control for assistive rehabilitation devices to all the experts and scientists. The third goal is to recognize the gap of knowledge that demands further investigation and to recommend directions for future research in this area.

## 1. Introduction

The aging population is considered a worldwide problem as the physical disabilities and weaknesses in elderly people have become a social problem in many countries [1]. Additionally, the number of senior citizens is dramatically increasing all over the world. It is observed that the estimated senior population percentage in Asia and in European countries is rising at an intensifying rate. Therefore, there is an immediate need to create assistive healthcare technological innovation to fulfill the needs of this significant portion of the community [2]. For instance, a research from the United Nations demonstrates that citizens aged 60 and above formed almost 11.5% of the global population in 2012, and this number is estimated to increase by 22% in 2050. Neuromuscular diseases such as stroke, spinal cord injuries, multiple sclerosis and weaknesses of the skeletal muscles seriously affect the day to day activities of senior citizens and patients. Hence, the quality and quantity of rehabilitation and training needed for the elderly, disabled, and those with other movement disorders needs to be increased.

Rehabilitation aims to restore the patient’s physical, neurological, and psychological abilities that were missing due to injury, sickness, and disease, and to support the affected person as a complementary for deficits that cannot be handled clinically [3]. Traditionally, this task was performed manually by therapists in rehabilitation centers or in hospitals. However, rehabilitation treatments are very work-intensive, especially for lower limb recovery which often demands more than three therapists together to support manually the legs and torso of the patient to perform the training [4]. Moreover, in a conventional manual therapy, the most suitable method that could be used to alleviate these conditions largely relies on the experience of the therapist, making it even more difficult to meet the requirements of certain forms of training that are considered to be repetitive with high-intensity. Hence, there is a demand to develop new therapeutic techniques and assistance methods that assist the elderly patients to boost their day to day activity performances and to recover the lost or impaired motion control. In addition, it helps to release the therapists from the intensive labor of rehabilitation training [5].

In the past decade, exoskeleton robotic devices (wearable robots) have been developed as a practical complementary system for therapists to handle impaired joints or limbs. Exoskeletons refer to a mechanical wearable device that is designed to mimic body parts such as ankle joints; when the device is worn, the torque produced by the actuators is transferred to the body [6]. Exoskeleton robot can efficiently incorporate the cognitive ability of a human being and the benefits of robotic techniques to assist users in performing the activities. These devices have been developed into full-limb exoskeletons, that is, upper and lower limb exoskeletons and other exoskeleton robots, to support shoulders, elbows, wrists, and ankle joints.

In addition to the development of sophisticated exoskeleton design, scientists also focus on helping the control techniques to enhance the accuracy, performance, and the comfort of the exoskeletons. Since exoskeletons are donned by humans, the interface between the wearable robot and the human is a vital factor to ensure smooth and effective control techniques that depend on the estimation of the user’s movement intention. Therefore, those control strategies can be classified according to the human-robot interaction method; according to the information obtained from the exoskeletons, based on the interaction force signal measured between the human and the exoskeleton and according to the physiological signal measured from the human body that indicates the user motion intention [7]. The assistive restoration improvement is enormously influenced by electrophysiological signals. These signals have been well-known tools to examine the capacity and conduct of the human movement in ongoing researches [8]. Electromyography (EMG) has been one of the frequently used physiological signals in the control techniques of exoskeleton systems because EMG can directly reflect a human movement intention or muscular action of the user [9]. On the other hand, with the latest improvements in technological innovation, brain computer interfaces (BCI) or brain-machine interface (BMI) have attracted a lot of focus in the bio-robotics area. Several techniques have been reported for capturing the brain activities. Among those techniques, electro-encephalography (EEG) is recognized as a non-invasive and a convenient method which may be appropriate for realistic applications.

Recently, it was found that less endeavors have been made to efficiently audit these reviews, as a way of offering analysts and specialists with a synopsis of the current, best-in-class EEG-based control systems utilized for assistive innovation. Hence, this research has three primary objectives. The primary aim is to deliberately assemble, abridge, assess and organize data with respect to accuracy and estimations of the past research distributed in the publications between 2011 and 2018. The second objective is to broadly report on all about the trial results of this domain’s present research. It is methodically performed to give a clear picture and grounded proof of the momentum conditions of research covering EEG-based control uses and benefits for controlling assistive robotics to every specialists and researchers [10,11,12,13]. The third objective is to perceive the whole of information that requests in-depth examination and to suggest ways for future research in this domain. To achieve these objectives, the following research questions (RQs) have been put forward:

(Q1) What are the types of EEG signals that are used to control the assistive devices?
(Q2) What are the different assistive devices?
(Q3) How do these signals translate to control commands?

The solutions to these questions will guide the reader and enhance their knowledge of the recent development of assistive robotics based on the EEG signals. A more extensive image of various emergent topics/themes, experiments and concepts will be offered. This paper is structured into six sections. The following section provides a background of EEG signals and assistive robotics. The third section describes the methodology through which the review processes were conducted. The fourth section presents the SLR results, followed by the fifth section which reports on the results of the research questions’ as organized according to their sequences. Finally, the sixth section presents a discussion of the review and its conclusion.

## 2. Background

### 2.1. Exoskeleton or Assistive Robotics

Robotic exoskeletons can be categorized into three wide categories according to their purpose. The first group is human efficiency enhancement exoskeletons that aim to maximize the durability, stamina, and other physical abilities by able-bodied persons. Augmentation exoskeletons might be employed for assisting with lifting heavy items, transporting heavy loads over long distances or handling heavy tools. The likely locations for these devices are manufacturing facilities, development sites, in urgent relief functions, or military bases and adventure activities. The second wide category involves assistive devices for people with movement disorders due to stroke, spinal cord injury and muscle weakness. Therapeutic exoskeletons are the third wide category and are utilized for rehabilitation purposes.

**Figure 1:**
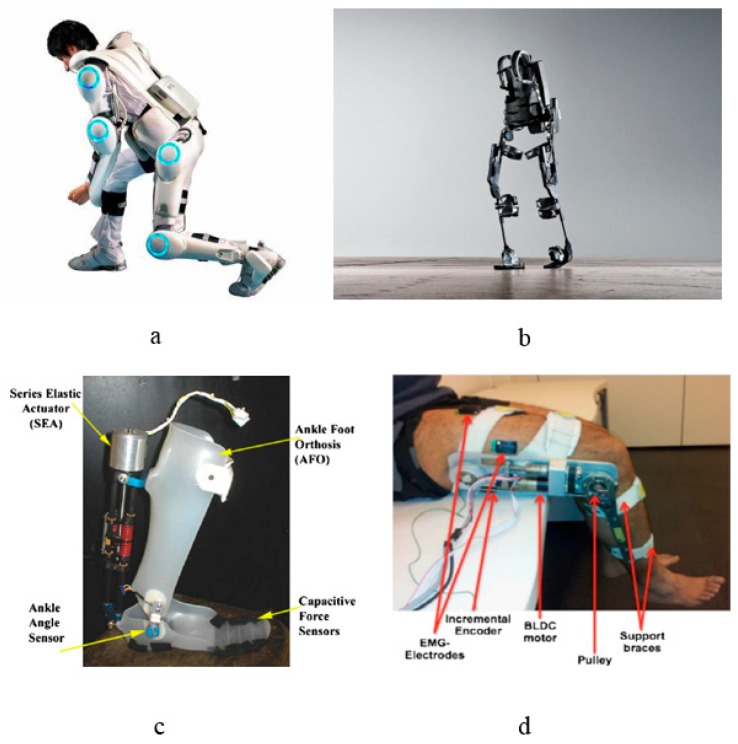
Exoskeleton Types (**a**) HAL 5 exoskeleton [17] (**b**) Ekso exoskeleton [17] (**c**) MIT AAFO [18] (**d**) Knee joint exoskeleton [19].

Based on the body part involved, robotic exoskeletons can be categorized into three different categories: upper limb, lower limb and specific joint exoskeletons [14,15,16] such as shoulder, elbow, knee, ankle, etc. Figure 1 shows different types of exoskeleton robot. One of the most significant hurdles to be alleviated in exoskeleton research is the human-robot interaction and control. Different techniques have been presented in the literature to manage the human-robot interaction.

The first technique is the control algorithm which is able to predict or follow the user’s intention according to the information obtained from the exoskeletons. This type of control has been applied to the BLEEX and it utilizes the information collected from the exoskeleton only to estimate the user motion intention. To get this information, sensors of force and torque are connected to the hip, knee and ankle joints to detect the force and torques signals imposed by the actuators and the user [18]. Two closed loops are required for this control algorithm; one represents how the user affects the exoskeleton and the other shows the effect of the actuator upon the exoskeleton.

The second control strategy, the control algorithm designed according to the interaction force measured through the deformation of an elastic transmission element or structure placed at the exoskeleton robot link [20]. A human-robot interface according to the forces has also been designed for HAL-5 that utilizes the floor reaction force (FRF) to estimate the motion intent. The FRF is employed to determine the position of the center of gravity that can be an efficient information for the intention estimation [21].

#### 2.1.1. Upper Limb Exoskeleton

Upper limb robotic devices can be categorized mainly into two categories: exoskeletons and prostheses. Exoskeletons or orthoses are a piece of orthopedic equipment that can be used to assist with the disabilities and recover the functions of a person or to enhance the functionality of the affected limbs, whereas, prostheses are artificial replacements that a person can wear in place of a missing body part. Different types of upper limb exoskeletons are deployed in the robotic-based rehabilitation purposes according to the functionality of the robotic devices. For instance, Trackhold [22] and ArmeoSpring [23] upper limb exoskeletons are used for upper limbs dynamic tracking and gravity compensation. Those passive robotic is integrated with virtual reality training applications to help a patient to perform and replicate activates of daily living (ADL).

#### 2.1.2. Lower Limb Exoskeleton

Lower limb exoskeleton mobilization is a mechatronic system which assists recovery of walking and standing. Those devices can be divided into two wide groups: human gait trainers, which are intended to recover walking pattern, and those devices were designed in different aspects include; over ground exoskeleton such as (e.g., EKSO, developed by Ekso Bionics, Richmond, CA, USA) or treadmill based exoskeleton like (e.g., LOKOMAT, developed by Hocoma, Zurich, Switzerland) and also signal joint exoskeleton robot (e.g., the Rutgers ankle robot, developed by The State University of New Jersey (New Brunswick, NJ, USA). Current effort has been developed to integrate two components, combining the ideas of walk training with that of passive joint motion (ANYMOV robotic bed, BTS Bioengineering, Milan, Italy). The compresence of the two training modalities should ideally cover the rehabilitation needs from the very acute phase to successive steps of recovery [24].

### 2.2. Electroencephalography (EEG)

Electroencephalography (EEG) is the most common brain signal that has been utilized in brain machine interface applications. This popularity is due to several facts: EEG signals are non-invasive, low cost, compatible, portable and have a high temporal resolution in comparison with other brainwave measurements such as electrocorticograms (ECoGs), magnetoencephalograms (MEGs), functional magnetic resonance imaging (fMRI) and near-infrared spectroscopy (fNIRS). Electroencephalography can be defined as the measurement of the electric brain activity caused by of the currents induced by neurons within the brain. The EEG signal can be detected in a non-invasive way by placing the electrode on the scalp [25]. This justifies why the EEG measurement is the most widespread brain activity measurement technique [26]. In addition, it is comparatively affordable and provides a high temporal resolution (about 1 ms). However, it has a weak signal and is prone to several artifacts and relatively poor spatial resolution [27]. In the EEG measurement, the detected waveforms reveal the cortical electrical activity. Signal intensity of EEG activity is often quite small and measured in microvolt (μV) range. The main EEG rhythms are classified based on the frequency range as alpha (α), beta (β), delta (δ), theta (θ) and gamma [22], as described in Table 1.

## 3. The Review Method

### 3.1. Search Method

**Figure 2:**
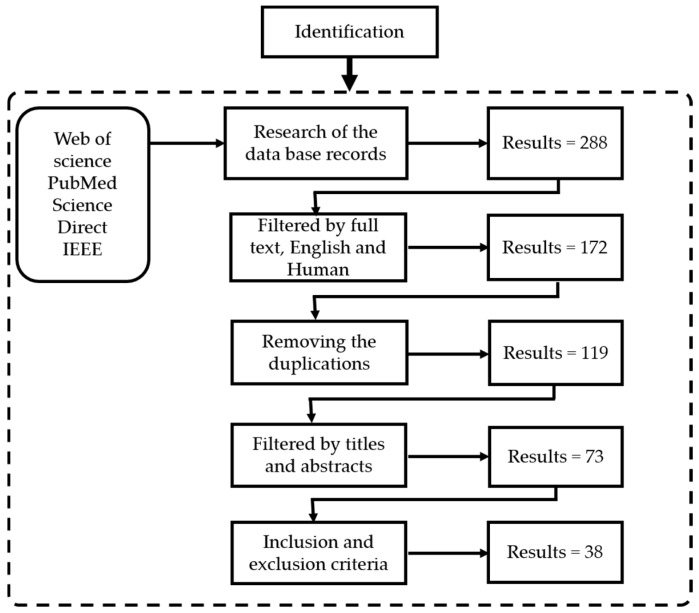
The search strategy.

As shown in Figure 2, an extensive literature search was carried out on PubMed, Web of Science, IEEE Explorer and on Science Direct. The search covered studies published between 2011 and 2018 and only the full-text reports published in English were considered. The combinations of search terms [(exoskeleton OR orthosis OR robot OR assistive OR prosthesis) AND (BCI OR brain computer interface OR BMI OR brain machine interface OR brain-controlled OR EEG OR electroencephalography) AND (Motor imagery OR Motor execution) AND (Lower limb OR Lower extremity OR Upper limb OR Upper extremity)] were used.

After carrying out the search procedures using the defined search-terms, this study identified 288 papers. Out of this number, 116 papers were filtered out by the criteria full text English and studies related to humans. The remaining 172 papers were duplicates and were appropriately removed through use of the Mendeley software. After the duplicate papers were removed, focusing on each paper’s title and abstract, 53 additional papers were removed. Then, the inclusion/exclusion criteria were applied to the remaining 73 papers. After reading the full text of the remaining papers, a total of 35 were excluded, leaving 38 papers for this research.

### 3.2. Data Extraction

From the selected research articles, general features were extracted, including the type of assistive robot that employed, the number of subjects recruited, the type of EEG signal used, if any other modality was involved such as EMG or EOG or other mechanical sensors, and their main outcomes. The authors accomplished individual assessments of the research abstracts and then determined which articles could possibly satisfy the inclusion considerations. For the articles that fulfilled the inclusion considerations, the full-text content material was obtained. The articles were classified according to the exoskeleton type such as the upper limb or lower limb exoskeleton.

## 4. Results

**Figure 3:**
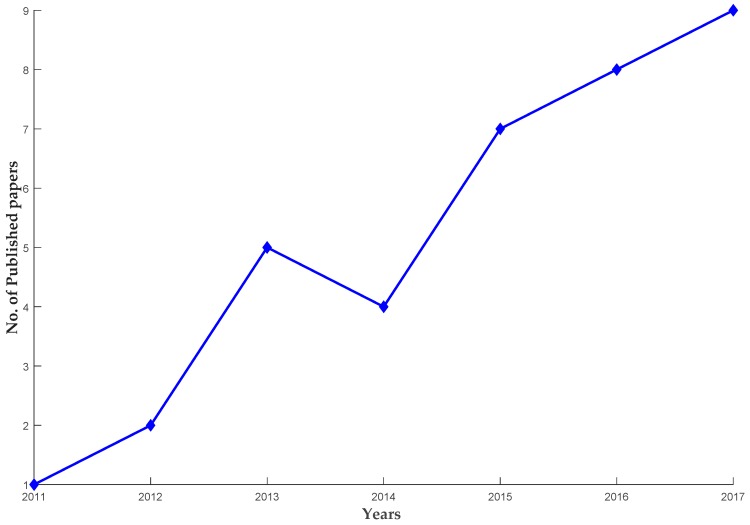
Publication distribution by year.

As a result of the systematic review, a total of 38 papers were finally chosen as principal studies, published within the field of research regarding EEG-based control for human limb exoskeleton as listed in Table 2 and Table 3. In order to emphasize more on how the EEG-based control field got more attention in the recent years, Figure 3 illustrates the chronological distribution of the selected papers in the interval of 2011 to 2017. The publication distribution shows the increase of the research papers with time, for instance, two papers were published in 2012, four papers in 2014 and the numbers dramatically increased from 2015–2017.

**Figure 4:**
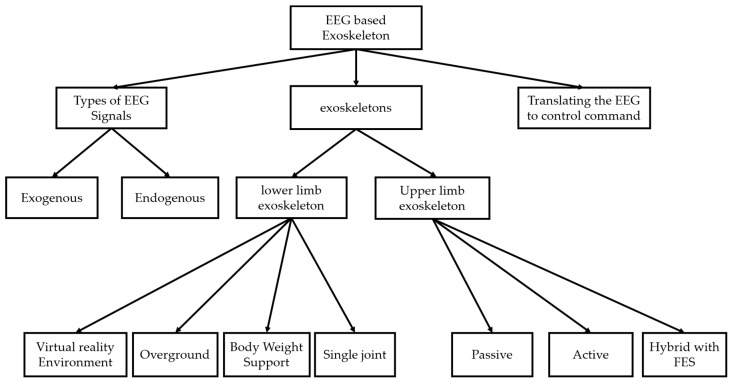
Research outcome.

The outcomes of this research are illustrated in Figure 4, where the research questions are addressed. Therefore, three key elements comprise the EEG-based exoskeletons include; types of EEG signals that measured in different tasks, types of exoskeleton devices in different aspects regarding to (the body parts and mechanical structure) and the interaction or translating the recorded EEG signal to a control command to manage the exoskeleton device.

### 4.1. Type of EEG Signal Used

**Figure 5:**
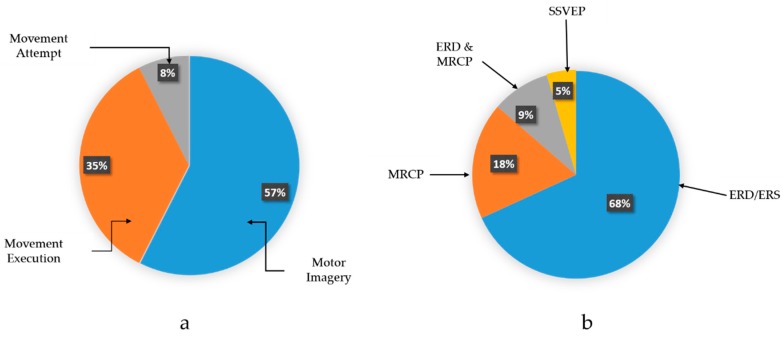
(**a**) Percentage of movement’s tasks and (**b**) Percentage of EEG signal type utilized in the selected studies.

Among the brain imaging modalities, EEG is still considered as the most realistic and practical non-invasive BMI technique nowadays. This is due to the fact that the other techniques like functional magnetic resonance imaging (fMRI), magnetoencephalography (MEG) and positron emission tomography (PET) are characterized by high cost, are not portable and are also technically challenging [30]. Therefore, according to the movements task, the assistive devices can be managed by utilizing exogenous (evoked) or endogenous (spontaneous) EEG signals. Figure 5a illustrates the different movement’s task that extracted from the selected studies in this survey.

#### 4.1.1. Exogenous EEG Signal

Using exogenous BMI, EEG signal is generated as a result of external stimuli such as auditory or visual clues. The benefits of this strategy involves minimal subject training, high bit rates up to 60 bits/min [56]. Nevertheless the subject required to always focus on the external cue or stimuli that restricts its applicability. Moreover, the subject can become easily exhausted as a result of the strong stimuli [30]. Steady state visually evoked potential (SSVEP) and P300-based interfaces are the examples of exogenous EEG signals [57].

SSVEPs can be defined as the natural reaction to the visual stimuli at varies frequencies [58]. In short, if one look at the flashing light with a specific frequency, visual cortex reacts with EEG signal at the same frequency. SSVEPs are utilized in exoskeleton robotics to send the control signal to the exoskeleton. The user is provided with possible control inputs on the screen such as move forward, move left and selects one by focusing at it. For instance, Kwak et al. in [34], implemented SSVEPs stimulation with visual stimulation screen consisting of five LEDs fixed to the exoskeleton. Each LED represent one control command (e.g., standing, walking forward, and turning right/left).

The P300 is another exogenous EEG signal type that appears around 300 ms after the user has noticed an external stimuli. Like the SSVEPs, P300 is implemented to choose one of numerous possible commands that the user intents will stimulate a P300 reaction. The P300 needs no training to implement, but has a lower data transfer rate than SSVEPs.

#### 4.1.2. Endogenous EEG Signal

On the other hand, in endogenous BMI strategy, EEG signals are produced independently from and external stimulation and can be completely managed voluntarily by the subject. Additionally it is practical for subjects who have neurological problems while offering a more natural and spontaneous way of interactions since subject can automatically control the neuroprosthesis [59]. It typically requires, however, longer training sessions and the bit rate is usually lower than those in SSVEPS and P300. A good examples of endogenous EEG signal include sensorimotor rhythms (SMR) and slow cortical potentials (SCP) [60]. Figure 5b shows the percentage of the endogenous or spontaneous EEG signal that employed in the selected studies in this review.

SMR can endure two kinds of amplitude modulations known as event-related desynchronization (ERD) and event-related synchronization (ERS). Sensorimotor rhythms consist of mu and beta rhythms, which are oscillations in the brain activity localized in the mu band (7–13 Hz) and beta band (13–30 Hz), respectively. ERD is indicated by a decrease in EEG power related with motion-related tasks in both active motion and motor imagery cases. Sensorimotor rhythms are relevant to motor imagery task without having any effective movement [61]. This increases the feasibility of utilizing sensorimotor rhythms for the design of endogenous BCIs, which are more beneficial than exogenous BCIs. This finding is consistent with the survey results where the 57% of the selected studies utilizing motor imagery task and ERD is the most common signal that have been used in the selected studies [12,23,24,29,40,42] to control the assistive devices as illustrated in Figure 5b.

Movement-related cortical potentials (MRCPs) reflect primary processes proportional to motor execution and are related to both active and imaginary motor task [22,47]. The dependence of MRCPs on force-related factors might be utilized to produce control signals for manipulating assistive robotics by disabled individuals. Xu et al. in [13], implemented MRCPs to detect the imaginary ankle dorsiflexion movements within a short latency from scalp EEG. Furthermore MRCPs can be integrated with other EEG modalities such as SMR as reported in [26,31], where the two modalities were integrated to control the ambulation of the lower limb exoskeleton during walking.

### 4.2. EEG Based Lower Limb Exoskeletons

Different types of EEG-based exoskeleton paradigms have been employed in the neurorehabilitation field including simulated avatars, body weight supported exoskeletons and over ground lower limb exoskeletons. Furthermore, walking training was achieved by deploying single joint assistive devices such as hip, knee, and ankle or ankle-foot orthoses.

#### 4.2.1. Virtual Reality Environments

Several studies have utilized brain control interface on the basis of EEG signal to control ambulation within a virtual reality environment (VRE) and consequently suggest that EEG based control for lower limb exoskeleton might be possible. To manipulate the linear ambulation of an avatar, five SCI participants carried out an online goal-oriented task. They deployed kinesthetic motor imagery (KMI) to control walking and idling state. Outcomes of classification accuracy of the different states i.e., idling and walking were predicted offline and varied from 60.5 to 92.3% for all participants. Moreover, the offline analysis demonstrated that the most salient feature for discriminating between walking and idling KMI was the activation of mid-frontal areas mainly in the μ and β bands. While in the online task the average performance was 7.4 ± 2.3 with successful stops in 273 ± 51 s. This study reveals that SCI patients are able to manipulate a self-paced BCI walking avatar to achieve goal-oriented ambulation task [35].

In addition, avatars or VREs have been used for the long-term training as a part of multistage BMI-based gait neurorehabilitation schemes targeted at recovering locomotion. Brain signals were recorded through 16 EEG channel to control the maneuver of the avatar body while receiving visual-tactile feedback [31]. Although VRE technology has been used in neurorehabilitation based on BMI to help in gait recovery, it has also been utilized to investigate the dynamics of cortical involvements in the individual treadmill walking. Luu et al. in [28], investigated the involvement of the cortical area in a human walking on a treadmill with and without closed loop EEG-based control. Substantial variations were discovered by source localization in cortical areas activity during walking with and without closed loop EEG-based control. The analysis results were as follows; in the Posterior Parietal Cortex and Inferior Parietal Lobe, maintained α/µ suppression was noticed, reflecting increases of cortical engagement in walking with EEG based control. Also a significant increase in the low-frequency band was seen in the anterior cingulate cortex (ACC), indicating the existence of cortical engagement in error monitoring and motor learning.

#### 4.2.2. Overground Lower Limb Exoskeleton

A closed loop EEG-based system to control a lower limb without any weight or balance support for gait rehabilitation to improve motor recovery in people suffering from the neuromuscular disease is reported in several research papers. Overground lower limb exoskeleton has been employed in the neurorehabilitation task to induce plastic changes in affected brain areas. López-Larraz et al. in [31], proposed a closed BMI to control overground lower limb exoskeleton for gait rehabilitation of SCI patient. This exoskeleton is a wearable with six degrees of freedom for the three lower limb joints include hip, knee and ankle joints. In addition, the exoskeleton control was developed to work under assistance as needed scheme to make rehabilitation more effective for the patients. The EEG signals of patients were employed to detect their gait intention and trigger the ambulatory exoskeleton’s movements.

Among the different types of the lower limb exoskeletons, Rex Exoskeleton is self-balancing which can achieve essential functions including walking front, back and side, sitting down and standing up, turning left and right. Lee et al. in [30], demonstrated online control of the modified Rex in the basis of EEG signals measured over the subject’s sensorimotor cortical networks. The classifier was trained to classify two different mental states that implemented in a cascaded way to drive the lower limb exoskeleton in three different ways such as walk front, turn left and turn right. The cascaded classifier was demonstrated by detecting a subject’s intention at the first stage to maintain walking front or turning the direction. Then, once the intention of the turning is detected, a binary decoding is achieved to move the exoskeleton left or right. The powerful use of cascade decoding for controlling exoskeleton is for performing high classification accuracy and reducing the mental burden of a user [30].

#### 4.2.3. Body Weight Supported Lower Limb Exoskeleton

Body weight supported lower limbs are a combination of a robotic gait orthosis and a body weight support system (BWS). This combination can be integrated with a treadmill such as Lokomat [33] or on the other hand integrated with overground lower limb exoskeleton as illustrated in the KineAssist robotic device [62].

Do et al. in [11], integrated BCI with a commercial lower limb exoskeleton RoGO system. In order to facilitate the integration of the EEG-driven RoGO, the EEG data were acquired from subjects as they involved in switching tasks of idling and walking KMI. This information was then analyzed off-line to develop an EEG prediction model for on the online BCI operation. Two metrics were evaluated to assess the performance of this system; cross-correlation and latency between the computerized cues and BCI-RoGO response. The offline accuracy of the EEG prediction model averaged 86.30% across both subjects. The cross-correlation between instructional cues and the BCI-RoGO walking epochs averaged across all subjects and all sessions were 0.812 ± 0.048.

In order to prove the concept of the feasibility of decoding the patient intention from the cortical network during the exoskeleton—assisted rehabilitation, García-Cossio et al. in [33] proposed a BCI robot-assisted paradigm. In this paradigm, a Lokmat Pro was employed as a walking assistance and is treadmill-based incorporated with BWS system. The user’s intention has been classified according to two walking phases; active and passive phases which are based on the detected EEG signal. The EEG signal was measured using 62 channel and the artifacts were removed using canonical correlation analysis CCA. Then the power spectrum analysis was evaluated using Welch’s paradigm with a Hanning window of 250 ms. In addition, power in frequency 8–30 Hz was normalized by the corresponding baseline condition to calculate the event-related desynchronization (ERD) and event-related synchronization (ERS) as follows:

$$\mathrm{ERD}\;\mathrm{or}\;\mathrm{ERS}=\left(\frac{\mathrm{Walking}-\mathrm{Baseline}\;}{\mathrm{Baseline}\;}\right)\times 100$$

The classification process was achieved by utilizing an L2-regularized logistic regression classifier. Classification accuracies for active and passive walking with baseline were 94.0 ± 5.4% and 93.1 ± 7.9%, respectively. While the accuracy between active and passive waling was 83.4 ± 7.4%. For stroke patients, the accuracy of walking with baseline was 89.9 ± 5.7%. This result revealed the viability of BCI—based exoskeleton assisted training device for walking rehabilitation.

By integrating two EEG modalities namely SMR and MRCPs, Liu et al. in [26] demonstrated an EEG-based brain-controlled lower-limb exoskeleton for gait training. The structure of the brain-controlled system consists of three layers; EEG decoding layers, interfacing layer and hardware implementation layer. EEG signals were recorded from the motor cortex area. Data collection, visualization, and synchronization were implemented using lab streaming layer. The customized exoskeleton consists of a serial-parallel dynamic mechanical structure which consists of 1-degree of freedom (DOF) hip joint and 2-DOF knee joint in the sagittal plane. The controller of the exoskeleton robot consists of a principal module (master) and a subordinate module (slave). The EEG signal was converted to control the robot through a transmission control protocol-internet protocol (TCP/IP). Moreover, the PID controller was implemented in an exoskeleton with closed-loop position feedback control. Using SMR-based method, the experimental results generated an average robot-control accuracy of 80.16 ± 5.44% and an average performance of 68.62 ± 8.55% with the MRCP-based method.

A multistage BMI system for gait neurorehabilitation based on EEG signals for motion recovery have been proposed in [32]. This system is a combination of intensive virtual reality training with visual-tactile feedback, and walking with two different EEG-based control exoskeletons which include a treadmill-based lower limb exoskeleton (LokomatPro, Hocoma, AG, Volketswil, Switzerland) and overground lower limb exoskeleton (ZeroG, Aretech, Ashburn, VA, USA). The subjects have been trained with this paradigm for 12 months duration. Thanks to the capability of the two EEG based control exoskeletons to provide tactile feedback to the subjects, all patients’ encountered neurological enhancements in somatic sensation in multiple dermatomes. Furthermore, a noticeable enhancement was seen in the patient movement’s index due to their ability to recover voluntary motor control in targeted muscles below the SCI level as measured by EMGs.

#### 4.2.4. Single Joint Exoskeleton

Neuromuscular disease can result in minimizing the activities of the muscles around the knee or ankle joints consequently causing the lack of ability for a person to lift a foot. Several techniques have been designed either in stationary or active foot orthosis to recover this ankle or knee mobility [4].

Therefore, ankle-foot orthosis has been implemented to restore the joint mobility and at the same time induce the neuroplasticity in the cortical areas. In the EEG based controlled lower exoskeleton field, Xu et al. in [13] implemented an ankle-foot orthosis which is developed for stroke rehabilitation. In this paradigm, imagery ankle dorsiflexion movements were measured within a short latency from the recorded EEG signal via the processing of MRCPs. The motor imagery was detected online using locality preserving projection method. Then each detection elicits a passive ankle joint dorsiflexion by triggering the motorized ankle-foot orthosis MAFO. The demonstrated MAFO-based EEG system offered a short latency and efficient technique for inducing neuroplasticity via BCI and has a good prospect in motor function rehabilitation after stroke.

Besides, imagined passive ankle joint movements also induce neuroplasticity changes. Central modulatory influences play an important role in the plasticity change and are utmost significant in rehabilitation [63,64]. Therefore the topological distribution of the ERD/ERS and task-related coherence during exoskeleton assisted and a motor imagery in ankle-foot movements has been investigated in [24] to give rich information of rehabilitation paradigm. A BTS ANYMOV robotic hospital bed was used to actuate ankle joint movements and 32 EEG were employed to record the brain activity during cyclic right ankle movement. Task-related coherence was evaluated in three different frequency ranges including alpha1 (8–10 Hz), alpha2 (10.5–12.5 Hz) and beta (13–30 Hz). The outcomes of this investigation were as follows; desynchronization in the alpha2 rhythm on C3 and ipsilateral frontal locations (F4, FC2, FC6) during the passive movements and beta ERD was recorded over (Cz, C3, C4). While during motor imagery task, a noticeable desynchronization was present for alpha1 over C3 and for alpha2 on (C3, C4). Moreover, task-related coherence reduced through passive movement in alph2 between contralateral central area (C3, CP5, CP1, P3) and ipsilateral frontal area (F8, FC6, T8); while the coherence of the beta band was diminished between C3–C4, and increased between C3–Cz. As a result, these quantitative measurements findings contribute to the understanding of the design and development of the neurorehabilitation methodologies.

### 4.3. EEG Based Upper Limb Exoskeletons

According to the survey results, upper limb exoskeletons based on EEG signals can be grouped into three different categories which include; active upper limb exoskeletons, passive upper limb exoskeletons, and integration of both exoskeleton types with functional electrical stimulation FES. Indeed, VRE has been employed in the passive upper limb exoskeleton to give feedback to the patient and allow the operators to evaluate the patient recovery progress as well.

#### 4.3.1. Passive Upper Limb Exoskeleton

Use of robotic systems for assisting the victims of neurological conditions is an approach that has already been suggested by [65,66] due to its capacity to improve the range of movement in limbs. Active systems of this kind that engage the patient in guided movement can potentially be unsafe in certain instances, with [67], pointing out the danger of overextending the limb. Meanwhile, it was demonstrated by [68] that passive devices complemented with VR capacity had the potential to be used for this purpose without the risks associated with active systems.

Steinisch et al. in [50] proposed an integrated passive robotic system for assistance in post-stroke recovery named Trackhold. It employs a passive robotic device in order to compensate the effects of gravitation and allow the exercise of the upper limbs while deploying multiple virtual engines to visualize motor tasks and facilitate interaction, along with an EEG-based system for monitoring of neural activity during exercise. Those three components are coordinated in real time, providing complete support for the patient during the rehabilitation process. The passive robotic device consists of three major parts. The first one is the end effector, which the patient holds in his hand and applies force. The second is armrest which can be configured for the left or right arm serves to provide stable support for the disabled limb. The final part includes the counter-weights, which serve to compensate for gravity and make the exercise less demanding for the patient. This is a completely passive device, which means the limb is never guided in the motions. Angular movement is registered by the sensors in the end effector, which feed the data into a 3D model where they are used to run virtual applications. A software solution with four classes of variables connects the robotic device with VR apps which the patient is logged into. A monitoring system based on high-resolution electroencephalography was implemented through 128 surface electrodes positioned in an elastic cap that measured neural activity during exercise. A signal amplification system was used for clearer recording of neural impulses. Data acquired in this way was divided into epochs and synchronized with inputs from the other two components, completing the picture of the patient’s activity on the level of his muscles, senses, and nerves. This allowed for neural reactions to be tied to individual events that occurred while limb activity was performed, providing direct insight into the progress of recovery. Findings from dynamic and EEG data evaluation illustrate the viability and prospective effectiveness of the suggested neurorehabilitation system to observe neuro-motor restoration.

Besides controlling the exoskeleton robot, EEG signals have a great impact in monitoring the brain function during the replication of active daily living. The proposed paradigm consists of passive upper limb exoskeleton robot called (Trackhold) which is used for dynamic tracking and gravity compensation, five specific virtual reality VR programs for the training of distinctive motion patterns and for cortical activity monitoring. In comparison with active upper limb orthosis, the Trackhold omits actuators for improved individual safety and satisfied levels, and for decreased complexity and costs. Findings from dynamic and EEG data evaluation illustrate the viability and prospective effectiveness of the suggested neurorehabilitation system to observe neuro-motor restoration.

#### 4.3.2. Active Upper Limb Exoskeleton

For upper limb robotic systems to be suitable for complex and precise tasks, the control interface needs to be very intuitive. Given that robot-assisted therapy can be very effective during post-stroke recovery, discovering better and less effort-intensive control mechanisms is one of the priorities in this research field. Providing a simple and effective means of neural control for robotic limbs is the step that could unlock the wider use of those precious therapeutic devices. This perhaps could lead to improvements in recovery rates for patients suffering from severe mobility issues. A robotic system that could be controlled with eye movement or motor imagery could conceivably be used in an everyday context for independent completion of basic tasks such as feeding, dressing or manipulating small objects.

Frisoli et al. in [54], presented a multimodal architecture which was proposed for control of a robotic arm. The system included multiple components which were connected into a unified control system that allowed subjects to complete common tasks such as reaching for various objects. Robotic exoskeleton attached to subject’s arm was capable of reaching and grasping, while the control was achieved through a chain of related mechanisms. BCI technology was used to capture neural signals, which were then decoded by an active vision system based on a Microsoft Kinect unit with an added eye-tracking device. An L-Exos exoskeleton for the right arm was used in the study, and it was chosen because of its lightweight and solid performance. Eye tracking system consisted of goggles equipped with infrared cameras, wide-angle cameras, and infrared LED lights. Since this device moves along with the subject’s head, it can be used to calculate the direction of his gaze in relation to the scene in front of him. The Kinect sensor serves as a target tracking device and enables precise object localization. One particularly important achievement of the proposed architecture is the availability of biofeedback from the robotic limb, making its use more natural and less mentally demanding. Based on the results of this study, it can be expected that fully functional BCI systems for exoskeleton manipulation will be developed along similar lines in the near future.

Cortical re-organization after exoskeleton-assisted training is a crucial factor that needs to be studied and understood. This could aid in improving rehabilitation therapy methods for stroke patients. Evaluation of cortical modification during passive robot-assisted motor movement, voluntary active movement in healthy subjects and motor imagery experiments were achieved under unimanual and bimanual methods in [51]. EEG signals were recorded with a video EEG technique in eight subjects during all the three motor paradigm tasks. The robotically assisted tasks were performed using a Bi-Manu Track robot-assisted arm trainer. The ERD/ERS method was implemented to demonstrate where movement-related decreases in alpha and beta power were localized. The findings of this investigation were as follows: on the contralateral side, a voluntary active unilateral hand movement was significantly observed, nevertheless, bilateral activation was observed in all participants on both the unilateral and bilateral active tasks. However a prevalent activation in the contralateral side was noticed during the passive motion when the right hand drove the left one. On the other hand, when the right hand is driven by the left one, activation was bilateral, particularly in the alpha band. Also, a considerable contralateral desynchronization was noticed during the unilateral task and ipsilateral ERD during the bimanual task.

Safety is an important factor in designing EEG-based control upper limb exoskeletons. An attainable approach to the enhanced safety of EEG-based assistive devices in daily life environments is the assistance as needed and switching off the BMI system when the brain activity is not required [69,70]. Furthermore, the integrated EEG signal with another physiological signal has been proposed to improve the BMI system safety and increase the degrees of freedom to manage the assistive devices. In this sense, Witkowski et al. in [48], presented an integration of EEG signals with electrooculography (EOG) signals to improve the reliability and safety of continuous hand exoskeleton-driven grasping motion. The proposed work implemented on conditions to control the hand exoskeleton includes; condition #1 using only EEG signal and condition#2 using fused EEG and EOG signals. Although all subjects participated in this experiment successfully performed control under both conditions including EOG, condition#2 significantly decreased unplanned hand exoskeleton movements.

Furthermore, a multimodal signal approach is reported by Kirchner et al. in [47] to improve the upper limb movements prediction performance and consequently enhance the control system of the external devices. The authors proved that an integration of EEG with EMG signals can effectively improve the versatility of assistive technological devices with respect to the persons’ requirements such as early and late phases in rehabilitation therapy. An AND combination was suggested, where two signals must be recorded to manage a movement. As a result, the behavior of the subject during late rehabilitation can be managed and also the false positive detection can be prevented. The final findings show that the classification accuracies of all modalities yield high performance in the range of 88–94%. The best result was yield from OR combination and EMG signal alone whereas slightly low accuracies were obtained from EEG mode alone.

**Figure 6:**
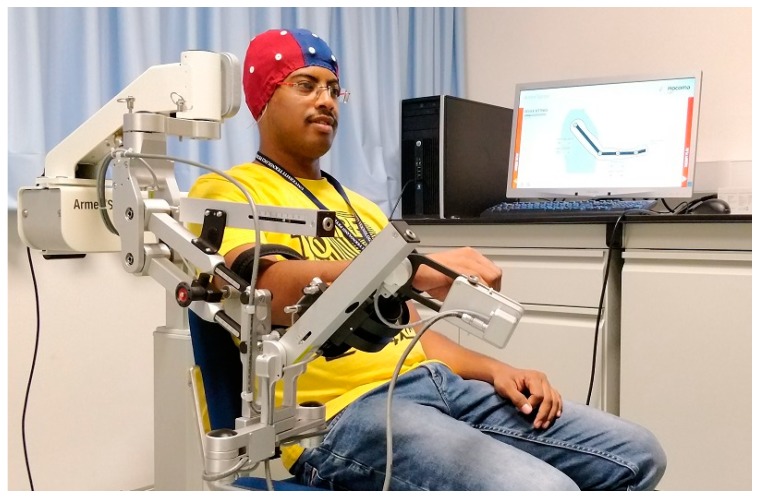
Upper limb (ArmeoSpring) at SMART Lab, UTP.

The feasibility of three-dimensional robotic assistance for upper limb reaching motions with a multi-joint exoskeleton during MI-ERD of sensorimotor oscillations in the β-band has been evaluated in [44]. In addition, task-related networks modifications of cortical activation connectivity in the basis of EEG were measured. In this experiment, the active upper limb exoskeleton (ArmeoPower, Hocoma, Volketswil, Switzerland) was employed. A similar setup but using ArmeoSpring at SMART Lab, UTP is shown in Figure 6. This robot is a multi-joint exoskeleton for shoulder, elbow and wrist joints with seven degrees of freedom. All participants including healthy and stroke survivors carried out their optimum performance during the initial phase of the experiment. Cortical network distribution of task-related coherence activity in the θ-range revealed a considerable difference between stroke patients and healthy subjects in addition to the variation in the late and early phases of the experiment.

User’s motion intention can be recognized from the cortical activity using EEG signal to control the external assisted devices for the daily activities. EEG based control is proposed by Tang et al. in [12], which states that modifications of the EEG can be valuable if self-induced as control commands for an upper-limb exoskeleton design by the authors. The classification performance of left versus right hand and left hand versus both feet by utilizing both motor execution (ME) motor imagery (MI) was assessed offline in the decoder-training stage. The outcomes showed that the classification accuracies of MI tasks were lower than those of ME tasks and also left hand versus both feet model obtained higher classification accuracy. Two conditions (wearing or without wearing the exoskeleton) the trained classifier were evaluated in the online-control session. The wearing the exoskeleton MI and ME session obtained classification accuracy of 84.29 ± 2.11% and 87.37 ± 3.06%, respectively.

The above-mentioned experiment in [12] has been carried out with healthy subjects, however; Bhagat et al. in [38], illustrated the feasibility of registering motor intent from cortical activities of chronic stroke patients by utilizing an asynchronous electroencephalography (EEG)-based brain-machine interface (BMI). User’s movements intention was predicted from MRCPs recorded over optimum EEG electrodes. Then, the upper limb exoskeleton (MAHI Exo-II) was triggered by successful intent detection to drive the upper limb movement and at the same time providing the user with a sensory feedback. The BMI training and optimization procedure was processed in 3 days. The BMI showed consistent performance with average true positive rate (TPR) = 62.7 ± 21.4% and 67.1 ± 14.6% on day 4 and day 5 respectively. Also, the average false positive rate (FPR) was 27.74 ± 37.46% and 27.5 ± 35.64% on day 4 and day 5, respectively. Overall, the motor intention was registered −367 ± 328 ms before movement onset during closed-loop BMI. The outcomes of this experiment revealed that the closed-loop EEG-based for stroke survivors, can be developed and optimized to carry out better performance across several days without system recalibration.

#### 4.3.3. Hybrid Upper Limb Exoskeleton with FES

Integrating upper limb exoskeleton and functional electrical stimulation (FES) were proposed to improve the effectiveness of rehabilitation outputs. Electrical stimulation of skeletal muscle groups has been applied as both a solely rehabilitation procedure to recover strength to atrophied muscles and to drive the paralyzed limbs of both stroke patients and tetraplegics [71,72].

Looned et al. in [49], presented an investigation through able body subjects which is the viability of assisting paralyzed patients through a mobile upper limb assistive technology. Authors in this experiment selected drinking a glass of water as a study task. To achieve this work, a combination of light upper limb robotic orthosis, FES, and wireless BCI on the basis of EEG signals were used. The robotic arm orthosis was designed to actuate the subject’s elbow in flexion/extension as well as forearm pronation/supination. In order to maintain the portability of the whole system, an EMOTIV EEG device which is a wireless headset was employed. The drinking experiment was divided into eleven phases out of which seven were performed by detecting EEG signal via BCI. FES system is integrated with robotic arm orthosis to support the hand in grasping/releasing an object. The use of FES is restricted by the hand movement only, which enables decreasing fatigue and also preserves the compactness of the whole system. All subjects performed the drinking task with an overall time of 127 ± 23 s.

To facilitate goal-oriented motor movement, Elnady et al. in [45], combined BCI based on EEG signals, FES and proprioceptive feedback. Moreover, the combination of these components was implemented to evaluate the feasibility of using the hybrid of EEG-based exoskeleton, and FES to assist motor task completion amongst people with stroke. The experiment was carried out with a subject with a chronic stroke (*n* = 9) and it was discovered that the training system was well accepted by all the participants. The results showed that the subject’s ability, to utilize EEG based driven exoskeleton with FES was not influenced by their ability to employ proprioception to control motor output.

In addition, passive upper limb exoskeleton was employed in a combination with FES to control an upper-limb in the basis of EEG signal. In this arena, Vidaurre et al. in [41] presented a motor imagery-based BCI to attain linear control of an upper-limb functional electrical stimulation (FES) controlled neuro-prosthesis. More in details, a passive upper limb exoskeleton (ArmoSpring, Hocoma) was used to compensate the arm weight and to permit the evaluation of the motion range and shoulder joint angle. The FES system controls the abduction and adduction of the arm through the motor imagery signal. Thereafter, the evaluation of the positioning precision and limb dynamics was conducted. The outcomes prove the feasibility of utilizing the BCI paradigm on the basis of EEG to manage limb movements.

### 4.4. Translating EEG Signal to Control Command

**Figure 7:**
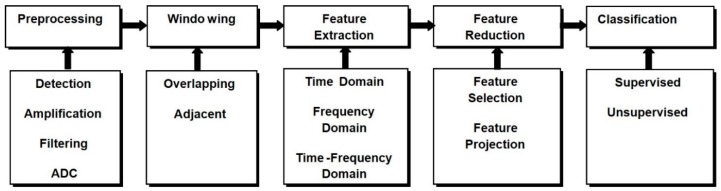
EEG signal decoding steps.

EEG decoding is a pattern-recognition method using digital signal processing and machine learning approaches. Thanks to advancements in technology, brain-machine interfaces (BMIs) have attracted a lot of interest in the bio-robotics area. These kinds of interfaces may open the routes to the straight decoding of the user’s brain signal to control devices such as exoskeletons as explained in the previous sections. These models allow the users whose neural system may have been destroyed by amputation, trauma or disease, to control an exoskeleton or any robotic device by decoding neurophysiological signals that are measured and processed in different steps as depicted in Figure 7.

The detected brain signal is pre-processed to remove the artifact to prepare the signal for the machine learning process that translates the EEG signals to the control command which drive the terminal devices such as lower limb exoskeleton. This process started with the feature extraction stage, and then the extracted features are subjected to the feature reduction technique if needed. Finally, the new projected feature vectors are classified into different classes according to the desired task. Therefore, to control external devices such as upper or lower limb exoskeleton in the basis of the EEG signal, the subject should generate different cortical activity patterns. The patterns include motor imagery [11,24,26,27,29] or motor execution [25,28,32,33], which will be recognized and translated into control commands. In the majority of current BCI, this depends on a classification algorithm [73], i.e., an algorithm developed to automatically predict the class of data as represented by a feature vector.

The feature extraction stage is the most significant part of the pattern recognition process mainly because useful information present in the raw EEG signal must be segregated and decoded for effective discrimination of the cortical activity by the classifier. Therefore, EEG feature extraction is a technique to transfer row input data into a diminished representation of a set of features which can be called a feature vector. An appropriate feature vector might as well hold convenient informative data and discard the irrelevant information. Different observable artifacts in the EEG measurement can be, such as eye movements, cardiac activity, and scalp muscle contraction [51]. The artifact removal was first applied to the raw EEG signal using different technique such as independent component analysis ICA [24], common spatial pattern (CSP) [54], canonical correlation analysis (CCA) [33], artefact subspace reconstruction (ASR) [28], common average reference (CAR) [30], z-scores [31]. Then the EEG data were segmented into windows which represented the class label or movement state such as idling and walking KMI states in [35], after which the features were extracted from each segment. According to the literature, different features methods have been implemented including time domain, frequency domain, and time-frequency domain.

Several decoders were employed in the selected studies and it can be classified into two categories according to the continual states being decoded [74]. The first category is characterized by continual estimation of trajectories, like unscented Kalman filter (UKF) [25,28] and linear regression (LR) [75]. The second, category is of binary classifier such as linear discriminant analysis (LDA) [13,23,26,27,40,42,44], support vector machine (SVM) [12,38,42,46,54,55], logistic regression [33], random forest (RF) [30] and neural network (NN) [55].

## 5. Discussion

Safety is a significant factor that should be taken into consideration when developing any lower limb exoskeleton. Therefore, comparing the interaction of the lower limb exoskeleton with the other assistive technology such as an upper limb, wheelchair or VRE, managing a lower limb exoskeleton or gait orthosis brings a significant risk to the subject safety [25]. Risks such as skin damage, falls, and bone fractures are involved in the expected risks of utilizing exoskeletons and this demands continued monitoring by research workers. Furthermore, EEG-based control for exoskeleton robots demands high-performance brain signal decoders, as any error can be costly when utilizing an exoskeleton. To overcome the expected risks, continuous monitoring from the researcher is needed, along with a design of the experimental protocol with a careful emphasis on safety. This is in reference to using BWS device to reduce fall risk such as treadmill based exoskeletons [33] or BWS with overground exoskeletons as reported in [11]. Nevertheless, BMI-based control of lower limb ambulatory exoskeletons demonstrated another problem in comparison with BWS exoskeleton. They needed to sustain balance by using parallel bars or by using a walker [31].

The outcomes of the research demonstrate the ability of the subjects with paraplegia or tetraplegia due to SCI to manipulate a non-invasive BCI-controlled avatar, a VRE, to achieve a goal-directed ambulation task [76]. However, some of the participants needed more training to attain BCI control. The benefit of using VRE with the SCI patients is to prepare the subjects for the active exoskeleton robot and this was proved by researchers in [11]. The performance of one subject with exoskeleton outperformed his performance with a BCI walking avatar in the prior study [76]. Furthermore, the investigation revealed that cortical network activities distribution varies from subject to subject during the walking KMI.

In addition, it has been proved that the gait assistance on the basis of the EEG signals is feasible [26,28,30,31], however, the level of assistance was varied between the healthy and SCI patient. In other words, the level of assistance provided in the SCI patient was higher than those in a healthy subject. This is because the SCI patient could not be able to generate the movements autonomously, in comparison with the healthy subjects who did not need to produce their high potential to follow the lower limb exoskeleton through its walking motion. However, this shows that the SCI patient can use their brain signal with less effort to drive or follow the exoskeleton robot even with a high support level. Consequently, these active participants would help to improve the neuroplasticity which could assist some level of motor recovery [26].

Effects of long-term BMI training on the basis of EEG signals has a significant impact on the patient sensation system and in regaining voluntary motor control in the targeted muscles as reported in [32]. The impact also extends to integrating the three different types of rehabilitation exoskeletons combined a VRE, visual-tactile feedback and walking with two EEG based exoskeletons including BWS and overground gait orthoses. Long-term BMI training is utilized on the basis of the EEG signal to improve the ability of the SCI patients to manage and drive the motion of the external assistive devices using their cortical activity. In addition to that, enhancing of neurological recovery is seen as a result of BMI training.

Type of EEG signals that are implemented in BCI is used as potential rehabilitation system. As illustrated in this survey, EEG-based control has been used to detect the cortical area activity such as the motor intention of the subject during motor imagination and consequently triggers the assistive devices or FES. Due to the fact that the distribution of the cortical activation throughout the MI is in the sensorimotor area, MI is sometimes named as sensorimotor rhythms (SMRs). SMR-based BCI was utilized to deliver visual feedback or to trigger assistive devices, such as lower limb exoskeleton [11,26,27,29,30], upper limb exoskeleton [42,45,49,52,54] or FES system [42,45,49]. Neuroplasticity occurs in both healthy or stroke subjects during the duration of immersive interventions which usually continued for several weeks and as a result insignificant plasticity was observed in the subject. However, short latency around 15 min intervention to induce considerable plasticity was reported in [13]. The reason behind the short latency is the implementation of MRCPs which is of high temporal precision.

Cortical activity distribution is important, to inform and help the developing of a rehabilitation paradigm. In the study achieved by [24], the results showed the quantitative measurements of the cortical activity throughout robot-assisted cyclic ankle motions. Brain activation of the motor cortex is indicated by alpha ERD, however, analyzing the afferent input of the somatosensory [77], common in the beta range plays a crucial role. Task-related coherence was evaluated in three different frequency ranges include alpha1 (8–10 Hz), alpha2 (10.5–12.5 Hz) and beta (13–30 Hz). The outcomes of this investigation were as follows; desynchronization in the alpha2 rhythm on C3 and ipsilateral frontal locations (F4, FC2, FC6) during the passive movements and beta ERD was recorded over (Cz, C3, C4). During motor imagery task, a noticeable desynchronization was noticed for alpha1 over C3 and for alpha2 on (C3, C4). Moreover, task-related coherence reduced through passive movement in alph2 between contralateral central area (C3, CP5, CP1, P3) and ipsilateral frontal area (F8, FC6, T8); while the coherence of the beta band was diminished between C3 and C4, and increased between C3 and Cz.

In addition, cortical modifications have also been evaluated in upper limb movements as reported in [51]. Three tasks were employed to assess the modifications of the cortical activations such as motor imagery, voluntary active movements, and passive robot-assisted movements bimanual and unimanual methods. ERD-ERS evaluation demonstrated the following—throughout the unilateral motion, bilateral activation was observed on SM1; although predominant contralateral activation was present, while the activation was located over the SM1c throughout passive unilateral motion and the imagination of unilateral motion. Considerable ERD was observed over SM1 (C3 and C4) during all bimanual motions. Moreover, during a passive robot-assisted motor performance, significant desynchronization of beta and alpha brain oscillations was recognized. Additionally, significant bilateral ERD over the sensorimotor areas was induced during bimanual passive robot-assisted movement. Finally, throughout the motor imagery experiments, the t-maps revealed the bilateral ERD on the bimanual task and contralateral ERD on the unilateral task.

Thus, exogenous EEG signals such as SSVEP and P300 require minimal training, have a high information transfer rate and also needs less electrodes on the scalp in comparison with SMR and MRCPs. However, both SSVEP and P300 required a screen attached to the exoskeleton and permanent focusing to the external stimuli that may cause tiredness and fatigue to some users. On the other hand, endogenous EEG signals are independent of any external stimulation, offer a more natural interaction and more suitable for patient with neural affected limbs. Nevertheless, endogenous EEG signals need long time for training, lower information transfer rate and also need more electrodes attached to the scalp.

A successful outcome of integrating gaze-BCI-based EEG signals has great implications for the future of robot-assisted rehabilitation. This is because it demonstrates the possibility to develop a simple and intuitive BCI-controlled system which even patients with serious disabilities can use on their own [54]. Selection of objects by gaze is probably one of the most natural-control mechanisms imaginable, while the motor imagery is a reliable technique for motoric activation. The multimodal framework proposed by the authors has practical advantages over existing models and offers more nuanced control of the limb, which is essential for both task execution and for robot-assisted rehabilitative exercise. The fact that the Microsoft Kinect system, which is commercially available and relatively affordable, is a crucial part of the system acts as a step towards standardization of BCI robot control. Based on the observed performance of the system, a practical gaze-driven therapeutic model could be developed if research in the same direction is pursued in the future. Application of this technology would greatly improve the outlook for patients in the earliest stages of post-stroke recovery since those individuals are having difficulty in communicating their intentions to move. With all this in mind, the study deserves to be followed up in a clinical setting to actualize full potentials of its scientific contributions.

Use of robotic devices during post-stroke therapy is already an accepted method, including passive devices similar to Trackhold. However, this idea is among the first to aim for full data integration and hence it sets an important cornerstone for future work in the technology-assisted stroke therapy. The inclusion of high-resolution EEG tracking and source localization of impulses were particularly innovative and potentially groundbreaking. Hardware and software components of the system were well coordinated. This was a very demanding task considering the complexity of the experiment and the number of parameters that had to be tracked. Interaction with VR application interfaces was very natural and intuitive thanks to video game-level quality of visualization, which is relevant for maintaining patient’s motivation for exercise and providing feedback that allows the patient to correct his movements. While the experimental group was too small to define reliable standards for the practical application of the device, the study nonetheless obtained critical data that will accelerate the pace of research in this field. The system is potentially compatible with other VR applications that require a planar workspace. However, it must be structurally altered in order to allow the exercise of various parts of the body other than the hand.

An important question has been raised regarding the effects of proprioceptive feedback on the regulation of cortical oscillations and consequently on the BCI control based on EEG signals. This question was addressed in [53], where the online BCI-based EEG was coupled with achand exoskeleton to control the flexion and extension of the fingers. Different tasks have been carried out with and without proprioceptive feedback. In some tasks, the SMR desynchronization was higher on average. This reflects a positive effect of proprioceptive feedback on cortical activity and on EEG based control performance. Nevertheless, insignificant difference was observed in the number of exoskeletons movement onsets between motor imagery with or without proprioceptive feedback in the contingent positive group. The two fundamental components of skill learning are not enough to enhance motor learning: learning without instantaneous rewarding feedback is not feasible and active-voluntary repeated actions alone will not be able to secure learning if it does not have feedback.

In order to leverage the effectiveness of neurorehabilitation therapy, a hybrid of upper limb exoskeleton and FES was proposed in [42,45,49], where the motor imagery was used to trigger the FES or exoskeleton. One parameter to evaluate the efficiency of the training device is the time consumed by the subjects to achieve a trial (Tc) [45]. Overall, the subjects were capable of completing three trials of the experiments. The value of Tc varied from 2.3 to 6.1 min and the average was 2.5 min. This mean value is actually quite acceptable with, i.e., roughly 2.2 min which is obtained when the system is perfect. Findings of this research are promising and also suggest that the individuals with expressive aphasia were capable to use the hybrid rehabilitation system. This illustrates the prospective utilization of the developed system in achieving goal-oriented motor task for multiple times in a 1 h rehabilitation intervention.

## 6. Challenges and Future Directions

Despite the rapid developments in EEG-based exoskeleton devices related to the upper and lower limbs, there are still several challenges associated to their real-time implementation. These open problems and challenges need to be addressed before these devices can be reliable, efficient, and affordable enough for patients to enhance the rehabilitation performance and quality of life. Some of these challenges are discussed in detail in the following paragraphs.

The major part of the recent research was forcing more in healthy subjects and some preliminary research proves the feasibility of implementing a non-invasive BCI-based control ambulation. Therefore, this could rationalize the upcoming progress of BCI-controlled lower extremity prostheses for free overground walking, for an individual with complete motor SCI. This includes overcoming the problem of increasing the degree of freedom for instance turning, sitting and standing. Since some of the studies use a relatively small sample (1 to 13), the reliability needs to be confirmed with further testing on both healthy and impaired individuals. Accuracy level achieved varies significantly from one individual to another, and is somewhat lower for stroke patients who would be the primary beneficiaries of a practical BCI-driven system. The system also can’t reliably differentiate between an intention to walk and actual muscular activity. Furthermore, the authors were unable to fully explain some of the differences between healthy and affected subjects regarding activity of the low gamma area, which leaves plenty of space for the follow-up studies to cover.

The difficulty in distinguishing more than three classes in real-time sessions of most BCI systems, on the basis of EEG systems, is the dominant challenge. Consequently, the reduced recognition accuracy in BCI systems with the addition of classes is another challenge. Furthermore, the design of reliable and robust BCI to identify brain signal related to the gait motion intention has not been so deeply examined when compared with upper-limb BCI. This is maybe the challenge of motion artifacts that associate the neural signal through the subject walking.

Moreover, another challenge facing the gait neural signal analyzing is the complexity of the set-up needed for control implementation. In addition, alternative and promising strategies for upcoming research could design a system that continually drives the external assistive robot movements, rather than detecting the intention of the movements and then triggering a predetermined trajectory. This should be the recommended way of assistive technology. It may also enhance recovery outputs by a more reliable organization between the paired firing of neurons, which may speed up the neuroplasticity changes [31].

Generally, the overall performance of the noninvasive BCI in EEG-based exoskeleton control is quite low in comparison to conventional control techniques based on mechanical or muscle activities [78,79]. This is applicable not only to the small variety of possible instructions per moment, but also to their characteristics, which is mainly digital (brainswitch). However, considerable utilization of shared control principles and context specific autonomy of the neuroprosthesis might help in compensating some of these limitations. Nevertheless, some issues continue to persist such as the need for calibration and adjusting due to the nonstationary characteristics of the EEG signals. The latency and low number of degrees of freedom of noninvasive BCIs are significant disadvantages for real-time, challenging exoskeleton control [80].

Moreover, in contrast with brain-controlled wheelchairs, lower extremity exoskeletons can be utilized not only to support subjects in ADL but also as training to induce neuroplasticity. However, different challenges need to be addressed. This includes a low value of SNR and motion artifacts that are included during exoskeleton-control stages; dysfunction like fatigue or loss of balance due to remaining in the upright position for a long time; the variation of the subject performance throughout the different sessions [26]. Hence, powerful artifacts removal model, additional support from a therapist and well-trained decoding algorithms should be integrated to alleviate these issues.

In summary, three significant gaps need to be addressed to make the EEG-based exoskeleton control ready for independent home use, these gaps include: (1) the usability; (2) the reliability; and (3) the translational gap [80]. Therefore, BCIs have to be enhanced to a level at which customers together with their care providers are able to implement the techniques individually at home. A key element for attaining this aim is the availability of easier to deal with, gel-less electrodes that provide an adequate indication quality. In order to demonstrate the reliability and usefulness of a BCI-controlled exoskeleton, long term studies with end users in real need to be performed. With the comprehensive execution of intelligent shared control techniques, uncertainties and non-stationarities, which are natural to non-invasive MI-BCI techniques, may be handled in part.

## Figures and Tables

**Table 1:** EEG Rhythms.

EEG Rhythm	Frequency Band (Hz)	
Delta (δ)	0.5–4	They appear in deep sleep and in infants.
Theta (θ)	4–8	They occur in the parietal and temporal areas in children.
Alpha (α)	8–13	They can be found in adults who are awake. These waves appear in the occipital area however, it can be detected in the parietal and frontal regions of the scalp.
Beta (β)	13–30	These waves are related to the movements and commonly appear in the frontal and central lope. The decreasing of the Beta rhythm indicates the movement, preparation of movements, planning a movement or imagining a movement [28]. This decrease is most dominant on the contralateral motor cortex. This attenuation in Beta waves is called event-related desynchronization [29]. The rhythms increase after the movement and are known as event-related synchronization.
Gamma (ɣ)	>30	It is the higher rhythms which has the frequencies more than 30 Hz.

**Table 2:** EEG-based control for lower limb movements.

[Ref] Year	Assistive Device	Participants	Protocol	Task	Control Input	Type of EEG Signal	Other Input Signals
[25], 2018	Avatar, BWS exoskeleton	8 healthy subjects	Active Movements	Gait	EEG based control		Goniometer
[26], 2017	Custom lower limb Exoskeleton	6 healthy subject	Motor imagery and movement intention	Gait	EEG based BCI	SMR and MRCP	Angle encoder
[27], 2017	RE lower limb exoskeleton	14 healthy subjects	Motor imagery	Rest/left and right hand	EEG based control	ERD	
[24], 2017	BTS ANYMOV robotic hospital bed	21 healthy subjects	Passive and imagined movements	Cyclic Ankle movements	EEG based control	ERD/ERS	
[28], 2017	Avatar	8 healthy subjects	Active Movements	Walking	EEG based control		Goniometer
[29], 2017	Prosthetic Knee	One amputee subject	Active movements	Sitting down and walking	EEG based control	ERD	
[30], 2017	The modified version of Rex (lower limb Exoskeleton)	5 healthy subjects	Motor imagery	Walk, turn right, turn left	EEG based control	ERD	Ultrasonic sensors
[31], 2016	Overground lower limb exoskeleton	3 healthy and 4 SCI patients	Movement attempt	Walking	EEG based control	Combination of ERD and MPCPs	
[32], 2016	Avatar, BWS and Overground exoskeleton	8 SCI patients	Motor Imagery and active movements	Gait	EEG based control	Event-Related Spectral Perturbations (ERSPs)	
[33], 2015	BWS Lokomat Pro gait Exoskeleton	10 healthy and three ISC	Motor execution	Active and Passive walking	EEG based BCI	ERD and ERS	EMG and accelerometer
[34], 2015	Overground exoskeleton	11 healthy subjects	Active movements	Walking, turn right/left	EEG based control	SSVEPs	
[13], 2014	Motorized Ankle-Foot Orthosis (MAFO)	10 healthy subjects	Motor imagery/active movements	Ankle dorsiflexion	EEG based control	MRCPs	EMG
[11], 2013	RoGO A commercial robotic gait Exoskeleton	On Healthy and one SCI subject	Kinaesthetic motor imagery (KMI)	Walking and Idling	EEG based BCI		EMG and Gyroscope to measure leg motion
[35], 2012	Avatar	5 SCI subjects	Motor imagery	Idling and walking	EEG based control		

**Table 3:** EEG based control for upper limb movements.

[Ref] Year	Assistive Device	Participants	Protocol	Task	Control Input	Type of EEG Signal	Other Input Signals
[36], 2018	Robotic Arm	19 healthy subjects	Active movements	Upper limb movement/reaching	EEG-based control	15–25 Hz EEG signals	
[37], 2017	Hand exoskeleton	64 stroke patients	Motor imagery	Hand open/closed	EEG-based control	5–30 Hz EEG signal	
[38],2016	MAHI exoskeleton	3 chronic stroke patients	Active movements	Elbow flexion/extension	EEG based control	MRCPs	EMG
[39], 2016	Prosthetic hand	2 amputee subjects	Motor imagery	Grasping objects	EEG based control	Low frequency-time domain feature	
[40], 2016	Arm exoskeleton	13 healthy subjects	Motor imagery	Reach and grasp tasks	EEG-based control	ERD/ERS	
[23], 2016	ArmeoSpring exoskeleton, Virtual arm and NMES	7 stroke patients	Active movements	Wrist extensor/flexor	EEG-based control	ERD	EMG
[41], 2016	ArmeoSpring and FES	7 healthy subjects	motor imagery	left hand, right hand, and feet	EEG-based control	7–30 Hz EEG signal	
[12], 2016	Custom upper limb exoskeleton	4 healthy subjects	motor imagery and motor execution	Left/right hand and left hand versus both feet	EEG-based control	ERD/ERS	
[42], 2015	passive exoskeleton ArmeoSpring and FES	3 healthy subject and 5 patients	motor imagery and movement intention	Arm reach movements	EEG-based control	ERD/ERS	
[43], 2015	Rhino XR-1 robot	30 subjects	motor imagery	left- or right-hand movements	EEG based control		
[44], 2015	ArmeoPower multi-joint exoskeleton	9 healthy subjects & 2 stroke patients	motor imagery	Arm reaching movements	EEG based control	ERD	
[45], 2015	Custom arm exoskeleton and FES	9 stroke subjects	motor imagery	A pre-defined goal-directed motor	EEG-based system		Joint angle encoder
[22], 2015	Upper limb exoskeleton	3 stroke patients	Active movements	Upper limb movements	EEG-based control	MRCPs	
[46], 2015	Hand exoskeleton	4 healthy subjects and one hand paralysis.	Active movements	Upper limb movements	EEG based control	ERD	EOG
[47], 2014	Upper limb exoskeleton	8 healthy subjects	Active movements	Upper limb movements	EEG based control	MRCPs (Readiness potential RP)	Eye tracking, EMG
[48], 2014	Hand exoskeleton	8 healthy subjects	Motor imagery	Hand movement	EEG-based control	ERD	EOG
[49], 2014	Lightweight Robotic Arm Orthosis (RAO) and FES	5 healthy subjects	Motor imagery	Assisting drinking	EEG based control	ERD	
[50], 2013	Trackhold upper limb exoskeleton	2 post-stroke patients	Active movements	Right/left arm movements		0.5–200 Hz EEG signals	
[51], 2013	Hand exoskeleton	8 healthy subjects	Active, passive and imaged movements	Hand movements	EEG-based control	ERD/ERS	
[52], 2013	MIT-Manus robot	6 stroke patients	Motor imagery	Upper limb movements	EEG-based control	4–45 Hz	
[53], 2012	Robotic hand exoskeleton	24 healthy subject	Motor imagery and active movements	Hand flexion/extension	EEG-based control	ERD/ERS	
[54], 2012	Arm Exoskeleton Light-Exos	3 healthy subjects and 4 patients	Motor imagery	Upper limb reaching movement	Multimodal (gaze-BCI based control)	ERD	Gaze tracker
[55], 2011	Robotic Arm	8 subjects	Motor imagery	Right/left upper limb movements	EEG-based control	Not specified	
